# Resolution of LPS-induced airway inflammation and goblet cell hyperplasia is independent of IL-18

**DOI:** 10.1186/1465-9921-8-24

**Published:** 2007-03-12

**Authors:** J Foster Harris, Jay Aden, C Rick Lyons, Yohannes Tesfaigzi

**Affiliations:** 1 Lovelace Respiratory Research Institute, Albuquerque, NM, USA; 2 University of New Mexico, Albuquerque, NM, USA

## Abstract

**Background:**

The resolution of inflammatory responses in the lung has not been described in detail and the role of specific cytokines influencing the resolution process is largely unknown.

**Methods:**

The present study was designed to describe the resolution of inflammation from 3 h through 90 d following an acute injury by a single intratracheal instillation of F344/N rats with LPS. We documented the inflammatory cell types and cytokines found in the bronchoalveolar lavage fluid (BALF), and epithelial changes in the axial airway and investigated whether IL-18 may play a role in the resolution process by reducing its levels with anti-IL-18 antibodies.

**Results:**

Three major stages of inflammation and resolution were observed in the BALF during the resolution. The first stage was characterized by PMNs that increased over 3 h to 1 d and decreased to background levels by d 6–8. The second stage of inflammation was characterized by macrophage influx reaching maximum numbers at d 6 and decreasing to background levels by d 40. A third stage of inflammation was observed for lymphocytes which were elevated over d 3–6. Interestingly, IL-18 and IL-9 levels in the BALF showed a cyclic pattern with peak levels at d 4, 8, and 16 while decreasing to background levels at d 1–2, 6, and 12. Depletion of IL-18 caused decreased PMN numbers at d 2, but no changes in inflammatory cell number or type at later time points.

**Conclusion:**

These data suggest that IL-18 plays a role in enhancing the LPS-induced neutrophilic inflammation of the lung, but does not affect the resolution of inflammation.

## Background

Processes involved in the initial generation of inflammation, i.e, infiltration of the lung air spaces by inflammatory cells and the associated changes in the airway epithelium, have been studied in great detail. However, only recent studies have focused on the resolution of inflammation that is generally characterized by a reduction in the number of inflammatory cells and the associated healing process of the airway epithelium. These studies have shown that the resolution of inflammation is not passive but an active and coordinated process with certain factors enhancing the resolution [1]. Understanding the events associated with normal resolution of acute airway inflammation could provide a basis for treatment and prevention of inflammatory diseases. Although several studies have focused on lipid mediators involved in the resolution of polymorphonuclear (PMN) cell influx and inflammation from various inflammatory insults [2,3], studies characterizing the resolution as a whole and cytokine patterns over longer periods after LPS-induced inflammation have not been reported.

Intratracheal instillation of LPS in the rat is designed to mimic the inflammatory response in patients with gram-negative bacterial infections. It is possible that aberrant repair processes are responsible for sustained pulmonary inflammation in the lung and airway remodeling observed in chronic diseases such as asthma and chronic bronchitis. Understanding the resolution process and factors that may be responsible for sustained inflammation or enhanced resolution are crucial to develop meaningful intervention strategies.

We have previously described the resolution of LPS-induced goblet cell metaplasia (GCM) in F344/N rats [4,5]. In order to identify possible mediators that affect the resolution of inflammation in the lung and thereby the factors that may affect the resolution of GCM we quantified inflammatory cells, major cytokines, and growth factors in the bronchoalveolar lavage fluid (BALF), and determined changes in the airway epithelium over a period of 90 d post-LPS instillation. Neutrophils [6], macrophages [7], and lymphocytes [8] have been shown to affect mucin expression and GCM directly or indirectly by modifying the presence of inflammatory mediators or affecting the resolution of inflammation. Therefore, we determined their numbers and the levels of inflammatory mediators during the course of resolution from an acute inflammatory response following LPS instillation. A 90-d study was selected to allow for the complete resolution of LPS-induced inflammatory cell influx.

This study showed that the resolution process is characterized by three stages of inflammation and demonstrated how the resolution of epithelial cell hyperplasia correlates with the resolution of inflammatory cells. IL-18 is a proinflammatory cytokine that can induce the p 38 MAP kinase pathway [9] and IFNγ-production in lymphocytes [10,11] and its levels showed a cyclic pattern over days 4–16. Despite its presence in the later stages of inflammation, reduction of IL-18 levels decreased neutrophilic inflammation at 2 d but did not affect infiltration of the lung by other inflammatory cell types or the resolution process following LPS instillation.

## Materials and methods

### Animals

Male pathogen-free F344/N rats (NCI-Frederick Cancer Research, Frederick, MD) were housed in pairs and provided food and water *ad libitum*. The rats were provided a 12:12-h light/dark cycle and an environment of 22°C and 30–40% humidity. Rats were randomly assigned to each experimental group, and were 9 wk of age at the beginning of this study. All animal experiments were carried out at Lovelace Respiratory Research Institute, a facility approved by the Association for the Assessment and Accreditation for Laboratory Care International.

### LPS-instillation and bronchoalveolar lavage

Rats were briefly anesthetized with 5% halothane in oxygen and nitrous oxide and instilled intratracheally (i.t.) with 1000 μg of LPS (*Pseudomonas aeruginosa *serotype 10, lot 31K4122, 3,000,000 endotoxin units [EU]/mg, Sigma-Aldrich, St. Louis, MO) in 0.5 ml of 0.9% pyrogen-free saline solution. Control rats were instilled with 0.5 ml of 0.9% pyrogen-free saline. Rats were sacrificed 3 h and d 1, 2, 3, 4, 6, 8, 12, 16, 40, and 90 post instillation with an injection of sodium pentobarbital and exsanguinated through the renal artery. Additional control groups of uninstilled naïve rats were sacrificed at the beginning and end of the study. The lungs were removed, lavaged and fixed as described previously. [12]

### Analysis of BALF

The total number of cells from the BALF were counted and the numbers of specific cell types were calculated as described previously [13]. The rat LINCO *plex *kit (LINCO research, Inc., St Charles, MO) was used according to package directions to determine levels of rat IL-1α, IL-1β, IL-2, IL-4, IL-5, IL-6, IL-10, IL-12, IL-18, IFNγ, TNF-α, macrophage chemoattractant protein-1 (MCP-1), granulocyte-macrophage colony-stimulating factor (GM-CSF), and Gro/KC (a chemoattractive factor). Levels were measured on a Luminex 100 system and data were analyzed using StatLIA software from Brendan Scientific (Grosse Point Farms, MI). Vascular endothelial growth factor (VEGF), insulin-like growth factor (IGF)-1, and IL-13 were measured with the R&D Systems, Inc. VEGF RatDuoSet (Minneapolis, MN), Octeia rat/mouse IGF-1 assay (Immunodiagnostic Systems, Fountain Hills, AZ), and the Biosource International Rat IL-13 ELISA (Camarillo, CA), respectively, according to manufacturer's directions. For graphical representation, values below detection limits were set to 0 pg/ml.

Because rat IL-9 was not commercially available, IL-9 levels in saline-and LPS-instilled rats were compared to uninstilled controls using an anti-human IL-9 antibody. BALF samples were plated in triplicate in wells of polyvinyl chloride, high-protein-binding, 96-well Costar plates (Corning-Incorporated Life Sciences, Acton, MA) and allowed to dry at 37°C overnight. The wells were blocked with PBS containing 1% normal goat serum for 45 min. Rabbit anti-human IL-9 antibody (Chemicon International, Inc., Temecula, CA) was diluted to 0.5 μg/ml in blocking solution, and the plates were incubated at 37°C for 2 h, then washed with PBS. A Vector Laboratories ABC kit (Burlington, Ontario) was used to detect the bound anti-IL-9 antibody, with the secondary antibody at a dilution of 1:200 in blocking solution and the ABC reagents prepared according to package directions. The horseradish peroxidase substrate tetramethylbenzidine was used to visualize the bound antiIL-9 antibody and was detected with a VERSAmax plate reader (Molecular Devices Corporation, Sunnyvale, CA) at 450 nm with a reduction at 650 nm.

### LPS quantification

The amount of LPS recovered in the BALF was assayed in duplicate using the Cambrex LAL Limulus Amoebocyte Assay (Walkersville, MD) according to package directions. Values are expressed in international endotoxin units (EU).

### Histology

The intrapulmonary airways of the left lung lobe from each animal were microdissected according to a previously described procedure [6]. Lung slices were embedded in paraffin, and 5-μm-thick sections were prepared for analysis of airway epithelia. Both the proximal and distal axial airway sections at generations 5 and 11, respectively, were analyzed for each of our data sets.

### Histochemical staining and analysis

Tissue sections were stained with Alcian blue, hemotoxylin and eosin (AB/H & E) as described [14]. Quantification of the total numbers of mucin-storing and non-mucin-storing epithelial cells was performed by a person unaware of slide identity using the National Institute of Health's image analysis system (Bethesda, MD) by counting the number of nuclei and dividing by the length of the basal lamina.

### IL-18 neutralization

Rats were anesthetized on day 0 as described above and i.t. instilled with 30 μg rat anti-mouse IL-18 (MBL Medical and Biological Laboratories, Nagoya, Japan) or with 30 μg rat IgG_1 _isotype control (R&D Systems) in 300 μl saline and returned to their cages. After 1 h, all rats were re-anesthetized and instilled with 1000 μg LPS. Rats received i.p. injections of 10 μg anti-IL-18 or IgG_1 _on days 1, 3, 5, and 7, and were sacrificed on days 2, 4, and 8, post LPS instillation as described above.

### Statistical methods

Numerical data were expressed as the mean group value ± SEM. Data were analyzed using the Statistical Analysis Software (SAS) from the SAS Institute, Inc. (Cary, NC). Results grouped by time point and dose were analyzed using a two-way analysis of variance (ANOVA); values that were considered significantly different from each other by ANOVA were further analyzed using a post-hoc Tukey's t test. Data having only two groups were analyzed using a Student's t test. The criterion for significant differences was *P *< 0.05 for all studies.

## Results

### Inflammatory cells in the BALF

**Figure 1:**
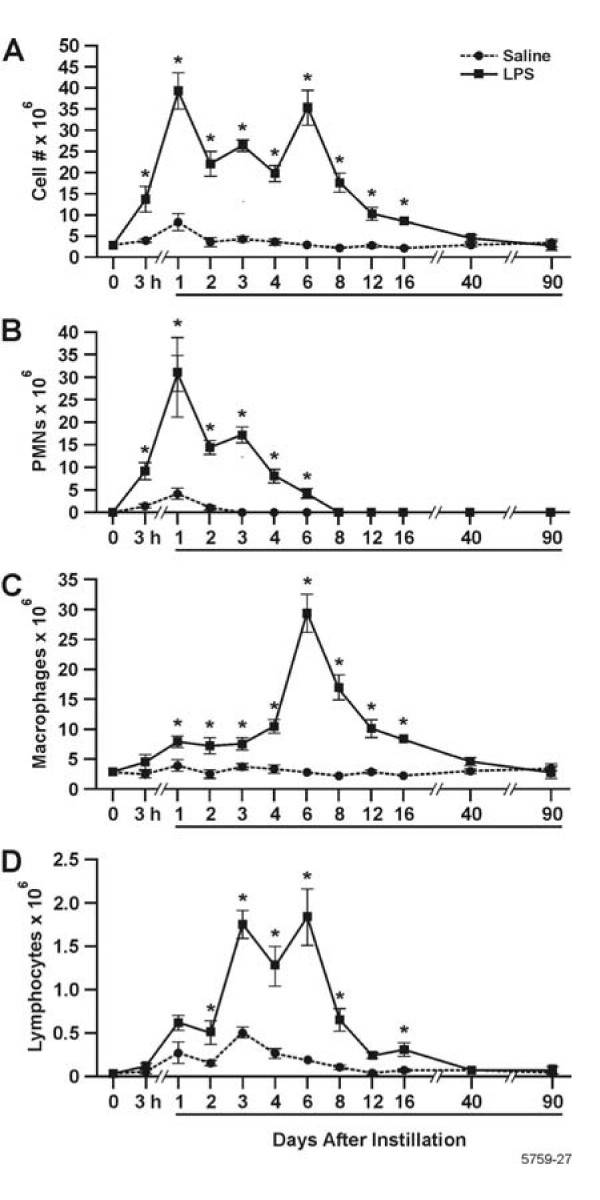
Three stages of inflammatory cell influx were identified in the BALF, characterized by PMNs, macrophages, and lymphocytes. White blood cells on cytospins were stained with Wright Giemsa and cell counts were performed. A: Total leukocytes; B Neutrophils; C:Macrophages; and D: Lymphocytes. Bars represent group mean values ± SEM (n = 5 rats per experimental group), * = statistically different from saline-instilled controls (*P *< 0.05).

The resolution of inflammatory cell influx into the lung was determined over a 90 d period post LPS instillation. The total inflammatory cells in the BALF reached maximum levels at d 1 and 6 post LPS instillation and returned to background levels by d 40 post instillation of LPS (Fig. 1A). The number of PMNs in the BALF was statistically increased compared to saline-instilled controls at 3 h, and reached maximum numbers at d 1, before dropping to control levels by d 8 post instillation (Fig. 1B). The number of macrophages in the BALF began to increase at 1 d, reached maximum levels at 6 d post instillation of LPS, and decreased over 40 d when they were no longer statistically significant from saline-instilled controls. (Fig. 1C). Lymphocyte numbers, although 10-fold lower than the numbers for PMNs and macrophages, reached maximum levels at d 3 through 6 and decreased to levels observed in saline-instilled controls at 40 d (Fig. 1D). Eosinophils were not present in the BALF.

### LPS and inflammatory factors in BALF

**Figure 2:**
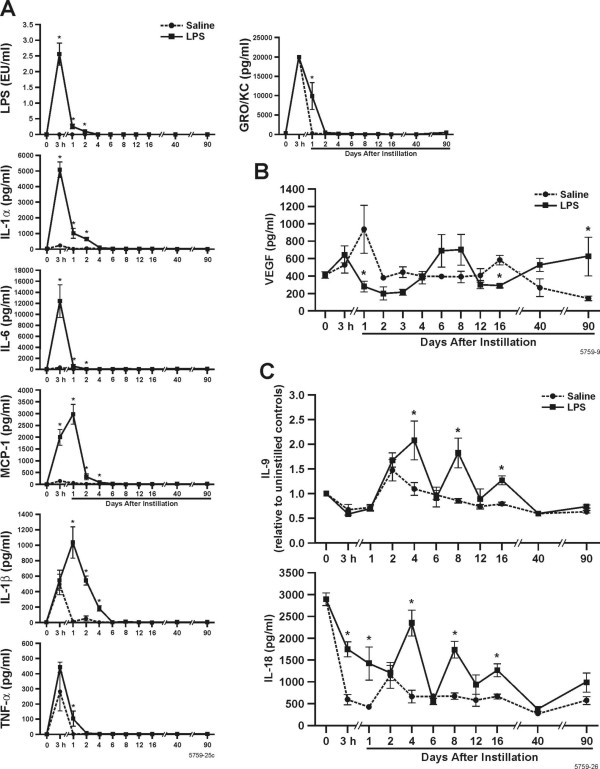
**Inflammatory mediators detected in the BALF**. A The amount of LPS recovered in the bronchoalveolar lavages was measured using the Limulus Amoebocyte Assay and expressed in international endotoxin units (EU/ml). BALF from rats instilled with saline or 1000 μg LPS were analyzed for cytokines and growth factors by multiplex ELISA and levels of IL-1α, IL-1β, IL-6, TNF-α, GRO-KC and MCP-1 over 90 d post instillation are show. B. VEGF was only increased at 8 d post- instillation of 1000 μg LPS compared to saline-instilled rats. C. IL-18 and IL-9 exhibit a cyclic pattern of expression over 90 d post instillation. Bars represent group mean values ± SEM (n = 5 rats per experimental group) * = statistically different from saline-instilled controls (*P *< 0.05).

The amount of LPS recovered in the BALF was highest at 3 h post-instillation and decreased to levels found in saline instilled controls by d 4 (Fig. 2A). We determined the levels of chemokines and cytokines in the BALF that have been reported to be important in recruiting and activating inflammatory cells to the airways and those that play a role in mucin synthesis and storage.

IL-1α (Fig. 2A) was highest at 3 h post-instillation of 1000 μg LPS, decreased to background levels by d 4, and remained low through 90 d. Saline-instilled controls had low levels of IL-1α and β at 3 h and became undetectable by d 4. Similar results were seen with IL-6 (Fig 2A). MCP-1 and IL-1β (Fig 2A) were highest at d 1 and returned to background levels by d 2 and 6 post instillation, respectively. GRO-KC and TNF-α (Fig. 2A), were both highest at 3 h post instillation, but LPS-instilled rats were not statistically different from saline-instilled controls. While both cytokines were reduced at day 1, the resolution of these cytokines was significantly delayed in LPS-instilled rats compared to saline-instilled controls. IL-2, IL-4, IL-5, IL-10, IL-12, IL-13, IFNγ, IGF-1, and GM-CSF were below the detection levels of our assays.

VEGF levels (Fig. 2B) were decreased over 3 d, increased over 4–6 d and were again significantly decreased compared to saline-instilled controls at d 16. However, VEGF increased again at 40 and 90 d to levels observed in rats at 0 d.

Interestingly, IL-9 and IL-18 showed a cyclical pattern during the resolution of inflammation. In LPS-instilled rats, both cytokines were at levels similar to saline-instilled rats at d 2, 6, 12, 40, and 90, but were significantly elevated at d 4, 8, and 16 post LPS instillation. IL-18 levels were high in non-instilled rats and showed an overall gradual decrease over 40 and 90 d (Fig. 2C). BALF from non-instilled rats the same age as our instilled rats at the 40 and 90 d time points showed that IL-18 decreases with age and was not statistically different from either rats instilled with saline or LPS at that time point (data not shown).

### Goblet cells and total epithelial cell number

We have previously shown that LPS instillation results in epithelial cell hyperplasia that is manifested as GCM [15]. To determine how the resolution of inflammation correlates with the resolution of GCM, we quantified mucus storing and non-mucus storing cells per millimeter basal lamina (BL) over 90 d post instillation. In this animal model GCM does not occur until d 2 post instillation [15]. Therefore, we excluded 3 h and 1 d from our histological quantifications. Morphometric results were similar in both proximal (airway generation 5) and distal (airway generation 11) airways.

**Figure 3:**
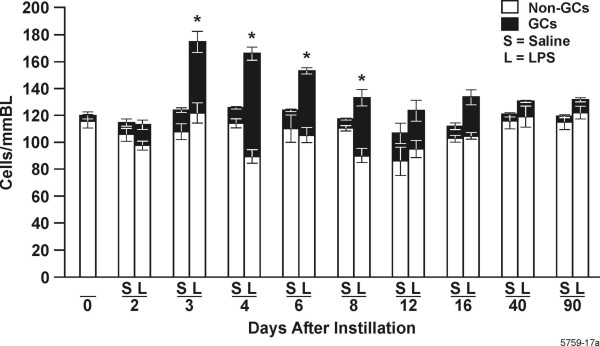
Total cell and goblet cell numbers reach maximum levels 4 d post instillation of 1000 μg LPS. Numbers of non-mucin storing epithelial cells/mmBL remain unchanged throughout the 90 d and are shown as white bars. Goblet cells per mmBL are shown as filled bars. Values given are ± SEM, (n = 5 rats per experimental group), * = statistically different from saline-instilled controls (*P *< 0.05).

Non-mucus cells per millimeter basal lamina (BL) remained statistically unchanged with approximately 90–120 cells/mmBL throughout the 90 d (Fig. 3). Rats instilled with 1000 μg LPS showed a significant increased number of total epithelial cells per mmBL compared to saline-instilled controls due to increase in mucous cells at d 3, 4, and 6. GCM declined significantly from 4 to 12 d, and remained at levels observed in saline-instilled controls through the 40-d time point.

### IL-I8 depletion

The cyclic pattern of IL-18 levels showed decreases at 6, 12, and 40 d post LPS instillation, when the numbers of PMNs, macrophages, and hyperplastic epithelial cells had declined to background levels. Therefore, we hypothesized that this cytokine may have a role in enhancing the resolution of inflammation and GCM. Studies have suggested that IL-18 may be associated with acute inflammation in the lung [16] or liver [17]. To test whether IL-18 directly affects the resolution of inflammation in our model, we depleted IL-18 in rats instilled with LPS and analyzed the inflammatory response at d 2, 4, and 8.

**Figure 4:**
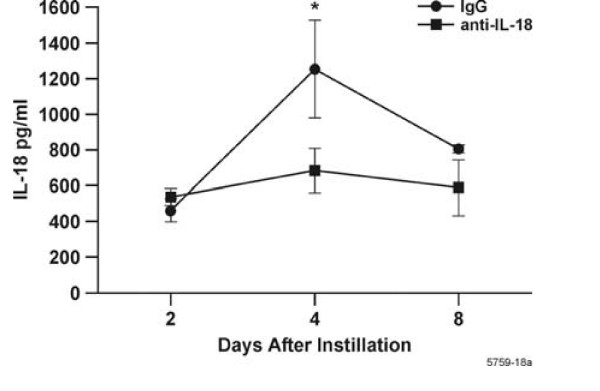
Reduction of IL-18 levels in the BALF of rats instilled and injected with anti IL-18 or control IgG_1_, then instilled with LPS and sacrificed 2, 4 and 8 d post instillation. BALF was obtained from IL-18-depleted and control rats as described above and IL-18 was determined using Luminex 100 technology. n = 3–6 rats per group. * = statistically different from controls treated with IgG_1 _(*P *< 0.05).

**Figure 5:**
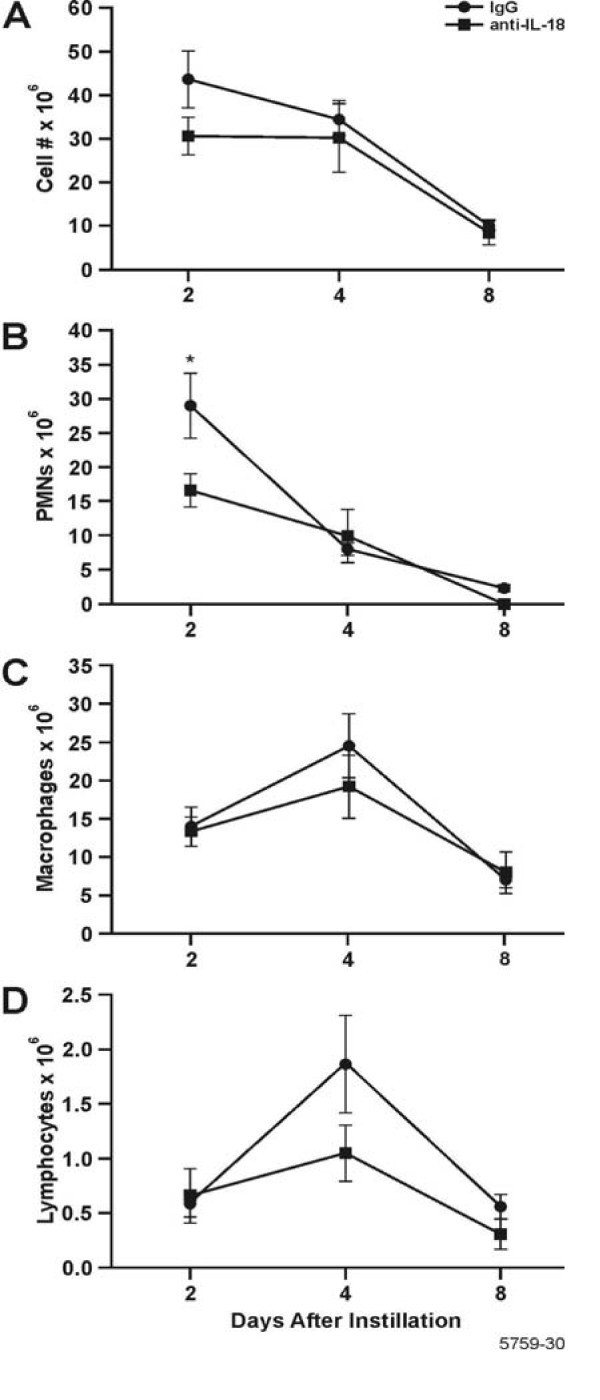
Inflammatory cell influx in BALF of rats instilled and injected with IL-18 neutralizing antibody and then instilled with LPS. Their lungs were lavaged and cytospin preparations were stained with Wright/Giemsa. The number of (A) Total leukocytes (B) PMNs; * = statistically different from controls treated with IgG_1 _(*P *< 0.05). (C) Macrophages;(D) Lymphocytes are shown. n = 3–6 rats per group.

**Figure 6:**
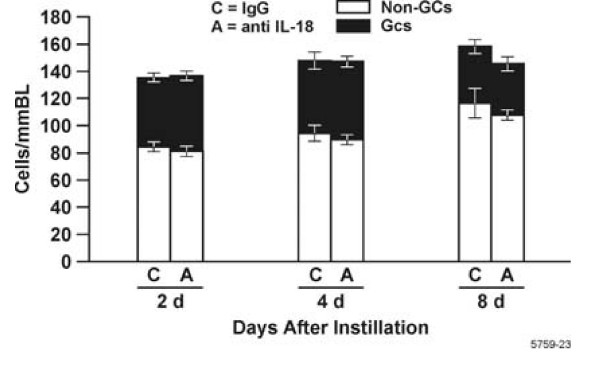
The number of total and metaplastic goblet cells in the airways of rats injected with IL-18 neutralizing antibody or IgG_1 _as control and then instilled with LPS. Non mucous cells are shown as white bars and goblet cells per mmBL are shown as filled bars. n = 3–6 rats per group

Injection with IL-18 antibodies reduced IL-18 levels in the BALF compared to IgG_1_-treated controls at 4 d post LPS instillation (Fig. 4). Interestingly, IL-18 levels in the BALF were unaffected at d 2 and 8. MCP-1, IL-1α, IL-1β and GRO-KC remained unchanged in treated animals compared to controls at all three time points (data not shown). Rats treated with anti-IL-18 and IgG_1 _did not exhibit differences in total numbers of leukocytes in the BALF (Fig. 5A) but showed a decrease in PMNs in the BALF at d 2 post LPS instillation (Fig 5B). The number of macrophages and lymphocytes remained unchanged compared to controls (Fig. 5C, D). LPS recovered by bronchoalveolar lavage was also unchanged in IL-18-depleted rats compared to controls (data not shown). Goblet cell, non-mucous cell and total epithelial cell numbers were not significantly increased in rats treated with anti-IL-18 compared to rats treated with control IgG_1 _at d 2, 4 or 8 (Fig. 6). However, both antibody treatments significantly increased total numbers of goblet cells at d 2 post instillation compared to rats instilled only with LPS.

## Discussion

The present study shows that following LPS-instillation, resolution of inflammatory cells and cytokines in the BALF is characterized by three different stages - influx of PMNs, macrophages, and lymphocytes into the lung airspaces. In addition, we show that IL-18 is not involved with the resolution process, but enhances influx of PMNs immediately after LPS instillation.

LPS-induced injury causes epithelial cells and macrophages [18,19] to produce C-X-C chemokines, such as GRO/KC (one of the murine IL-8 homologues) that attract neutrophils to the airspaces [20]. Therefore, neutrophils are the first cells at the scene following LPS instillation [3,21,22] and may be associated with the clearance of LPS [23] as shown by our finding that LPS in the BALF was reduced to background levels within 2 days. Removal of the initial stimulus, i.e. LPS, is critical, because persistence of offending agent could lead to chronic and ongoing inflammation.

The appearance of IL-1β, IL-1α, IL-6, and TNF-α, and MCP-1 in the BALF correlates with the first stage. The C-C chemokines such as MIP-1 are predominantly chemoattractants for monocytes [24] and the cytokines including TNFα, IL-1α and β and IL-6 can activate macrophages [25]. Because neutrophils are short-lived and undergo apoptosis within hours of entering the airspaces [26,27] activated macrophages must be present at increased numbers to enhance the capacity to clear apoptotic neutrophils by phagocytosis [28,29]. Therefore, the appearance of macrophages in the BALF defines the second stage. The clearance of inflammatory cells is also among the first steps in resolving the inflammatory responses [30,31].

Our observation that VEGF levels were decreased over 72 h post LPS instillation are consistent with other reports [32]. VEGF production peaks several days after injury in various systems [33] and is important in the resolution of inflammation in some tissues, because this process is characterized by enhanced angiogenesis [34]. The early production of angiogenic factors, such as TNFα followed by release of VEGF is believed to allow the formation of blood vessels that provide nutrients to tissues and allow trafficking of immune cells. Furthermore, recent studies suggest that dendritic cells when matured in the presence of anti-inflammatory molecules secrete VEGF and promote angiogenesis [35].

We found maximum numbers of macrophages at 6 d post LPS instillation, the same time point when increases in VEGF levels were detected in LPS- compared to saline-instilled rats. VEGF can be produced by various cell types [36,37], and our findings indicate that macrophages may be the main source for VEGF in the BALF at d 6 and 8 after LPS instillation. However, airway epithelial cells can also express VEGF when treated with IL-1β, TNFα, or neutrophil elastase [37]. Therefore, increase of IL-1β, TNFα, and other mediators at early time points following LPS-instillation may have initiated production of VEGF at d 6–8 post instillation.

The number of lymphocytes reaches maximum at 3–6 d characterizing the third stage of inflammation and is still 10-fold lower than the number of PMNs and macrophages when they reach peak levels. T lymphocytes may be the primary source of IL-9 [38] and IL-18 [39] while IL-9 can also be produced by neutrophils in the lung [38]. IL-9 and IL-18 levels in the BALF decreased as non-instilled, saline-instilled or LPS-instilled rats aged over 90 d. However, in LPS-instilled rats, their levels decreased initially then increased in a cyclic manner at d 4, 8, and 16. These cyclic increases and decreases in IL-9 and IL-18 spanned all 3 stages of inflammation. To our knowledge, such cyclic pattern has not been reported during resolution of acute inflammation.

We tested the lavage fluid for the known anti-inflammatory cytokine, IL-10, assuming that this cytokine may be crucial in the resolution process. However, this cytokine was below detection levels (1 pg/ml). The other cytokine that is associated with resolution of inflammation is TGF-β. While several studies that have exposed rodents to LPS and reported that they did not detect increased TGFβ levels in the lung tissues [40,41] apoptotic cell recognition by activated macrophages and clearance induces TGF-β1 secretion resulting in accelerated resolution of inflammation [42]. Whether active or latent forms of TGF-β1 enhance resolution LPS-induced inflammation will be studied in the future.

The onset of GCM correlates with the decline of PMN numbers as was shown in previous studies [12,6,15], and is maintained through d 6 when macrophage numbers are highest. We also observed that macrophage and PMN numbers were equal and lymphocyte numbers were maximum by d 4 when GCM is highest, suggesting that a combination of factors derived from these cell types, may be contributing to epithelial cell hyperplasia and increased mucin synthesis and storage resulting in GCM. It is established that IL-6, produced by both macrophages and neutrophils [21], induces mucin synthesis [43]. IL-1β is produced by both PMNs [21] and lymphocytes [22] and can activate the MUC5AC promoter [44] in primary airway epithelial cells. Furthermore, neutrophil elastase prolongs the half-life of MUC5AC mRNA [45] and also leads to increased mucin expression through the generation of reactive oxygen species [46]. Increased IL-9 levels at 4 and 8 d correlate with increased GCM and are consistent with studies showing that IL-9 is necessary for the development of GCM [16,47,48]. GCM following acute injury or inflammatory responses results from differentiation of pre-existing epithelial cells into mucous cells and differentiation of proliferating cells to mucous cells [15,49]. Therefore, the inflammatory mediators may cause epithelial cell proliferation and directly induce mucin synthesis in pre-existing and proliferating epithelial cells *in vivo*.

When the number of mucous cells/mm basal lamina is subtracted from the total epithelial cell number at each time point, no major changes were observed during the resolution, indicating that the increase in total epithelial cell numbers is entirely composed of hyperplastic mucous cells. The reduction in the number of mucus-producing cells coincides with the total removal of PMNs and is associated with the decline of macrophage and lymphocyte numbers in the lavage fluid. The resolution of GCM involves various mechanisms. First, some of the mucous cells appear to transdifferentiate into non-mucus cells. This change must involve reducing mucus synthesis and possibly differentiating into ciliated [50] or serous cells (personal unpublished observation). This process of transdifferentiation could be due to the decline in cytokines and other inflammatory mediators responsible for mucin gene expression and the presence of a combination of inflammatory mediators stimulating the differentiation of these cells into another epithelial cell phenotype. Second, the resolution of GCM involves the reduction of approximately 30% of airway epithelial cells being removed from the epithelium. Because all of these cells represent mucus-producing cells, this mechanism may account for the reduction of at least 1/3 of mucus production. Our previous studies have shown that Bcl-2, an anti-apoptotic protein, is expressed in metaplastic mucous cells of LPS-instilled rats [4,15]. This resolution is at least in part orchestrated by Bcl-2 being downregulated allowing the pro-apoptotic members to elicit cell death and reduce the number of hyperplastic epithelial cells [5,51]. Whether the decline in specific cytokine levels causes downregulation of Bcl-2 expression and thereby the cell death of hyperplastic epithelial cells is being investigated.

**Figure 7:**
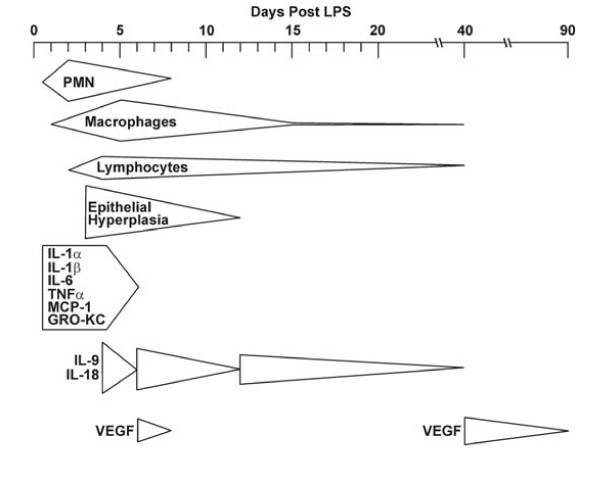
Overview showing the correlation of cells and cytokines during the resolution of inflammation after a single LPS challenge.

IL-18 in the BALF showed a cyclic pattern decreasing to background levels on d 6, when PMNs and the early cytokines had declined, then on d 12, when GCM was resolved, and lastly on d 40, when the macrophage and lymphocyte numbers had declined to background levels (Fig. 7). Because the role of IL-18 is largely unknown and we observed an unusual cyclic pattern of this cytokine during the resolution process we hypothesized that this cytokine may have importance in regulating resolution. Rats instilled with IgG_1 _showed increased GCM already at 2 d post LPS instillation while GCM was not observed in rats instilled with LPS only, suggesting that the antibody itself enhanced LPS-induced inflammation as is manifested by the doubling in the number of PMNs in LPS-instilled rats treated with IgG_1 _compared to those instilled with LPS only. Treating a group of rats with a 60-μg instillation followed by LPS instillation and 20-μg injections of anti-IL-18 or IgG_1 _as control caused such an increase in lung inflammation that the axial airway epithelium was completely denuded at 4 d post instillation (data not shown). Therefore, we determined that a smaller dose of antibody that maintained airway structure was more appropriate for our study. Furthermore, the total cell numbers of inflammatory cells at days 2 and 4 d was lower in LPS instilled rats compared to those instilled with LPS and treated with IgG_1 _and anti-IL-18 antibodies. While the numbers of macrophages and lymphocytes remained similar, increased influx of PMNs in anti-IgG_1_-treated rats likely caused GCM to increase earlier than in rats instilled with LPS only. Consistent with these findings, previous studies have documented that GCM can be reduced by depleting PMNs [6,12]. The lack of resolution of GCM at d 8 post LPS instillation in both the IgG_1_- and anti-IL-18-treated groups may be due to sustained changes within the airway epithelia because of elevated levels of cytokines.

The time points 2, 4, and 8 d post instillation were chosen for studying the effect of IL-18 depletion because major changes associated with resolution of inflammation were observed in the lungs of LPS-instilled rats at those time points: At d 2, PMN numbers in the BALF were reduced to 50% of their maximum; at d 4, GCM had reached maximum and PMN and macrophage numbers detected in the BALF were equal; at d 8, macrophage numbers were reduced to 50% of their maximum and GCM was reduced compared to maximum levels by d 8.

Our initial treatment with anti-IL-18 by i.t. instillation only did not reduce IL-18 levels in the BALF; therefore, we administered anti-IL-18 i.p. in addition to the i.t. instillation. Interestingly, IgG_1 _itself reduced LPS-induced IL-18 levels by one half, and this decrease was consistent throughout the time course studied. While anti-IL-18 was administered before and after LPS instillation, we found significant reduction in IL-18 levels only on d 4; why IL-18 levels were not reduced on d 2 post LPS instillation may be due to anti-IL-18 levels not having reached maximum levels at the early time point. Furthermore, compensatory mechanisms may have overruled the anti-IL-8 effect at d 8, reducing the difference to non-significant levels.

It is possible that the anti-IL18 IgG could have in-specific effects and neutralize other inflammatory factors. However, because the detected levels for MCP-1, IL-1α, and IL-1β were unchanged in anti-IL-18-treated rats compared to controls, we do not believe that the presence of anti-IL-18 antibody in the lavage could have affected the detection of cytokines. Reduction of IL-18 in the BALF and serum of rats treated with anti-IL-18 antibodies caused a significant decrease in pro-inflammatory serum cytokines such as IL-12, IL-6, and IFNγ at d 4 post LPS instillation compared to controls (data not shown). These findings suggest a role for IL-18 in the balance of cytokines in the blood during lung inflammation and resolution.

IL-18 depletion of LPS-instilled rats decreased the number of PMNs in the BALF at an early time point (2 d) compared to the IgG_1_-treated rats. Our findings are consistent with a short-term study of acute lung inflammation showing that IL-18 enhances PMN migration into the lung [16]. Studies of cystic fibrosis patients positive for *Pseudomonas aeruginosa *showed that BALF and leukocytes obtained from the BALF exhibit decreased levels of IL-18 compared to healthy control patients [52,53]. However, because IL-18 levels in CF tissues are higher than in control tissues, it is believed that the IL-18 detection in CF lavage is compromised by an unknown factor masking its detection [53]. In another study, pretreatment of allergen-sensitized mice with anti-IL-18 followed by an allergen challenge decreased PMN influx initially, but did not affect tissue inflammation, numbers of PMNs, or GCM at later time points. The lack of increased cytokine levels or leukocyte numbers in the BALF despite increased PMN numbers in anti-IL-18-treated rats suggests that there may be compensatory mechanisms maintaining the cytokine response to LPS.

The observation that IL-18 levels decreased immediately after LPS instillation when PMN influx was highest appears somewhat contradictory since treatment with anti-IL-18 antibodies reduced PMN numbers in the BALF at d 2 compared to IgG_1 _treatment. However, these findings suggest that the elevated amount of IL-18 present in the lung before LPS instillation may be crucial to the PMN influx and the decline of IL-18 from 3 h through d 2 in the LPS-challenged lung may prevent excessive neutrophilic inflammation and airway damage.

In summary, the present study shows that LPS-induced airway inflammation follows classic stages of resolution for PMNs, macrophages, lymphocytes and certain cytokines, but includes patterns of inflammation for IL-9 and IL-18 that have not been reported previously. Reduction of IL-18 reduced PMN influx at d 2 post instillation but did not affect the resolution process. We have reported essentially the same initial responses over d 2–4 to various lots of LPS that were prepared at different times either from *P. aeruginosa *[12] or from *E. coli *[15]. Therefore, this highly reproducible model system is useful to elucidate the mechanisms involved in the resolution process, to identify which inflammatory mediators may enhance the resolution of inflammation, and the elimination of hyperplastic epithelial cells along with the reversion of metaplastic mucous cells.

## Competing interests

The author(s) declare that they have no competing interests.

## Authors' contributions

JFH carried out the experimental procedures and prepared the general outline of the manuscript, JA analyzed the data statistically, CL was involved in the cytokine analyses, and YT conceived of the study, participated in its design and coordination, analyzed the data, and finalized the manuscript. All authors read and approved the final manuscript.
